# Model for estimating the population prevalence of chronic obstructive pulmonary disease: cross sectional data from the Health Survey for England

**DOI:** 10.1186/1478-7954-5-8

**Published:** 2007-09-26

**Authors:** Luis C Nacul, Michael Soljak, Tom Meade

**Affiliations:** 1 Health Protection Agency, 7th Floor Holborn Gate, 330 High Holborn, WC1V 7PP London, UK; 2 Public Health Information & Intelligence Strategy, Health Improvement Directorate, Department of Health, Skipton House, 80 London Road, SE1 6LH London, UK; 3 Department of Epidemiology and Population Health, London School of Hygiene and Tropical Medicine, Keppel Street, WC1E 7HT London, UK

## Abstract

**Background:**

Chronic obstructive pulmonary disease (COPD) is a major but neglected public health problem. Currently 1.4% of the England population has a clinical diagnosis of COPD, but the true burden of the disease has not been known with certainty, as many cases remain undiagnosed.

**Methods:**

A mathematical model based on cross sectional data from a representative sample of the population in England (the Heath Survey for England 2001, n = 10,750) was developed allowing estimates on the prevalence of COPD (defined based on the presence of airflow obstruction) to be obtained. Logistic regression analysis was used to investigate and choose risk factors for inclusion in the model and to derive the prevalence estimates based on the strength of association between selected risk factors and the outcome COPD. The model allows the prevalence to be estimated in populations at national level and also at regional and large local areas, based on their compositions according to age, sex, smoking and ethnicity, and on area degrees of urbanisation and deprivation. We applied the model to measure the prevalence of COPD in England and in some sub-groups of the population within the country.

**Results:**

The prevalence of COPD in England is estimated as 3.1% (3.9% in men and 2.4% in women) in the population over 15 years of age, and 5.3% (6.8% in men and 3.9% in women) in 45 year-olds and over. There was a 7-fold variation in the prevalence across subgroups of the population, with lowest values in Asian women from wealthy rural areas (1.7%), and highest in black men from deprived urban areas (12.5%).

**Conclusion:**

The model can be used to estimate population prevalence of COPD from large general practices to national level, and as a tool to identify areas of high levels of unmet needs for COPD priority health actions. The results from the model highlight the importance of including variables other than age, sex and smoking, i.e. levels of deprivation, urbanisation and ethnicity, when estimating population prevalence of COPD. The model should be validated at local level and incorporated into case-finding strategies.

## Background

Chronic obstructive pulmonary disease (COPD) is a chronic condition characterised by progressive airflow obstruction, which is not completely reversible. COPD accounts for nearly 30,000 deaths each year in the United Kingdom (UK), corresponding to 5.7 percent of adult male and 4 percent of adult female deaths, including a significant number of premature deaths. In addition, 1.4% of the population consult their general practitioners (GPs) for COPD each year. It accounts for 2% of hospital admission spells and over 3 percent of bed-days in adults [1], costing the NHS £800 million, and leading to 24 million working days lost each year [2].

As expected, the prevalence of COPD is higher in smokers and in men, and it increases with age. Other risk factors of public health importance include air pollution [3], socio-economic deprivation [3], occupational exposures [3,4] and possibly ethnicity [5-7]. There is considerable variation in the reported prevalence of COPD.

Models using smoking rates to estimate COPD prevalence have been previously proposed [8-10], but none has direct relevance to the UK. We therefore developed a model (Model-HSE) to estimate the prevalence of COPD based on existing data from the Health Survey for England (HSE) 2001 [11], which has nevertheless not been used for this purpose before. It uses the main risk factors for COPD reported in the literature, particularly those that are easily measured and for which information is readily available. This report explains how the model was developed, and uses the population of England to illustrate its application.

## Methods

### Data sources

The distribution of COPD in the population of England was based on the HSE 2001 findings for lung function parameters and their association with relevant risk factors. The methods used in the HSE 2001 are described in detail elsewhere [11]. In brief, the survey included a representative sample of the population who had their lung function evaluated using a portable spirometer with a calibration device (Vitalograph 'Escort Spirometer'). Comprehensive data on risk factors were also collected as part of the survey. The data refer to 5,269 men and 6,133 women over 15 years old with valid lung function measures. This corresponds to 98% of men and 95% of women visited by a research nurse as part of the survey. For 99.3% of these, data were available for all of age-group, smoking status, ethnicity and degree of urbanisation. Data were available for deprivation score and all of the above risk factors in 94.3% of the final sample, which was used for the multivariate analysis. This included 4,970 men and 5779 women.

COPD was defined according to British Thoracic Society (BTS) criteria [12], based on the values of forced expiratory volume in 1 second (FEV_1_) and the forced vital capacity (FVC) i.e. FEV_1_/FVC < 0.70 and FEV_1_<80% predicted using British reference values derived from the HSE [13].

### Model construction

The choice of variables for inclusion in the model was based on logistic regression analysis that examined predictors of COPD using the HSE 2001 dataset. Explanatory variables obtained from the HSE dataset and included in the final model were (categories of each variable shown in brackets): gender, age group (15–34 year olds, 10 year age groups from 35 up to 74 years of age and 75 year olds and over), smoking status (smoker, former smoker, never smoker), ethnicity (White, Black or Black British, and Asian or Asian British), area of residence (rural, urban and suburban) and area based index of deprivation (quintile of deprivation score based on Index of Multiple Deprivation[14]).

The *baseline odds *of COPD (in non-smokers under 35 years old) were obtained directly from the data. Separate baseline odds were estimated for each gender, and also according to ethnicity, area of residence and area-based deprivation score.

The logistic model was used to derive the *odds ratios and prevalence ratios *for COPD for subjects with various combinations of risk factors in relation to baseline. The prevalence in each age group, gender, ethnic group, area of residence and level of deprivation, and smoking status category were derived from the odds, using the formula: *Prevalence = odds/(1 + odds)*.

### Model application

The *input variables*, which could be defined by the relevant user, e.g. at Primary Care Trust, include age-group, gender, smoking prevalence by gender and age, area of residence, area based deprivation score and ethnic distribution of the population.

### Model outputs

The main model outputs are the prevalence of COPD by gender for the relevant geographic area, at national, regional or local level, as defined by the user. To illustrate the use of the model, we have used here population inputs for England, based on the mid-2005 estimated population distribution [15] and the national smoking prevalence by age-group and gender for 2004–2005 [16].

### Model assumptions

The model assumptions include: i) The real prevalence of COPD in non-smokers under 35 years of age (baseline prevalence) is the same as the prevalence in non-smokers of the same age group and gender in the 2001 HSE population; ii) The ratio of odds and prevalence of COPD in the various age groups compared to the baseline group is the same as in the HSE for each gender, smoking status and other risk factors in the model; iii) the risks in those falling within each of the risk categories are uniform.

We also obtained the prevalence of COPD considering alternative scenarios, which assume that: a) the prevalence of COPD in under 35s or under 40s is uniform across smoking status in each gender, and is equal to the average baseline prevalence found at the HSE (therefore it does not consider any increase in risk due to smoking in this age group); or b) the prevalence of COPD is zero in under 35s or in under 40s; or c) ethnicity does not have an effect on the risk of COPD, and the risks in white populations apply to all ethnic groups.

## Results

### Risk factors for COPD and selection of variables for Model-HSE

Table 1 shows the results of the univariate and final regression logistic models assessing risk factors for COPD. Risk of COPD is significantly lower in women than in men (odds ratio (OR) = 0.64; 95% CI = 0.54 – 0.76; p < 0.001). As gender was shown to modify the effect of other variables on the outcome, the analyses were carried out separately for men and women. No other significant interactions were found in the data, including between age-group and smoking status.

**Table 1:** Risk factors for COPD included in the final prevalence model

Variable	Odds Ratio (95% CI) (univariate model)	Odds Ratio (95% CI) (final logistic model)
**MEN (n = 4970)**		

Smoking Status	(*P *< 0.001)	(*P *< 0.001)
- Never smoker	1	1
- Former smoker	3.63 (2.54 – 5.21)	2.18 (1.48 – 3.23)
- Current Smoker	3.81 (2.64 – 5.52)	4.50 (3.01 – 6.74)

Age-group (in years of age)	(*P *< 0.001)	(*P *< 0.001)
- <35	1	1
- 35–44	1.65 (0.86 – 3.17)	1.94 (0.99 – 3.78)
- 45–54	2.45 (1.33 – 4.50)	2.66 (1.41 – 4.99)
- 55–64	6.91 (4.02 – 11.89)	7.92 (4.46 – 14.07)
- 65–74	10.40 (6.08 – 17.80)	12.69 (7.12 – 22.60)
- 75+	12.15 (6.78 – 21.76)	16.02 (8.57 – 29.94)

Area of residence	(*P *= 0.02)	(*P *= 0.25)
- Urban	1	1
- Suburban	0.70 (0.50 – 0.97)	0.74 (0.51 – 1.06)
- Rural	0.58 (0.39 – 0.86)	0.72 (0.46 – 1.15)

Ethnicity	(*P *= 0.64)	(*P *= 0.95)
- White	1	1
- Black/Black British	1.20 (0.48 – 2.99)	1.17 (0.44 – 3.10)
- Asian/Asian British	0.69 (0.28 – 1.70)	0.97 (0.37 – 2.51)

Quintile of Multiple Deprivation Score	1.22 (1.11 – 1.34) (*P *< 0.001)	1.17 (1.05 – 1.31) (*P *= 0.005)

**WOMEN (n = 5779)**		

Smoking Status	(*P *< 0.001)	(*P *< 0.001)
- Never smoker	1	1
- Former smoker	1.70 (1.07 – 2.64)	1.26 (0.79 – 2.01)
- Current Smoker	3.53 (2.43 – 5.14)	4.11 (2.74 – 6.15)

Age-group (in years of age)	(*P *< 0.001)	(*P *< 0.001)
- <35	1	1
- 35–44	1.08 (0.53 – 2.19)	1.35 (0.65 – 2.79)
- 45–54	2.34 (1.28 – 4.30)	2.69 (1.42 – 5.09)
- 55–64	4.18 (2.34 – 7.47)	6.03 (3.26 – 11.15)
- 65–74	5.36 (2.99 – 9.61)	8.04 (4.33 – 14.91)
- 75+	5.34 (2.79 – 10.22)	10.40 (5.18 – 20.87)

Area of residence	(*P *= 0.01)	(*P *= 0.04)
- Urban	1	1
- Suburban	0.62 (0.42 – 0.92)	0.59 (0.39 – 0.90)
- Rural	0.47 (0.29 – 0.77)	0.55 (0.32 – 0.96)

Ethnicity	(*P *= 0.19)	(*P *= 0.50)
- White	1	1
- Black/Black British	1.14 (0.42 – 3.12)	1.81 (0.64 – 5.14)
- Asian/Asian British	0.24 (0.03 – 1.76)	0.60 (0.08 – 4.47)

Quintile of Multiple Deprivation Score	1.18 (1.05 – 1.34) (*P *= 0.005)	1.11 (0.98 – 1.27) (*P *= 0.11)

The final model shows age group and smoking history are the strongest predictors of COPD in both genders. Residence in urban areas and possibly black ethnicity are also associated with increased risk, particularly in women. Living in more deprived areas is associated with increased risk in men, but not in women. Being Asian appears to be protective in women, although this association did not reach statistical significance.

### Application of HSE-model to England population

**Figure 1:**
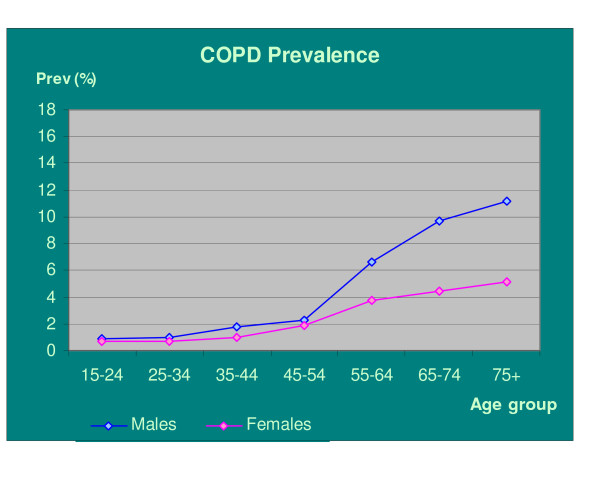
Estimated real prevalence of COPD in England population of median deprivation score by age-group and gender.

Table 2 and Figure 1 show the prevalence of COPD by age and gender in England. The overall prevalence in the population over 15 years of age was 3.1% (3.9% in men and 2.4% in women). For those over 45 years old, the estimated prevalence was 5.3% (6.8% and 3.9% in men and women respectively). This corresponds to over 1.3 million people in England with COPD, of whom nearly 800 thousand or 60% are men.

**Table 2:** Number and proportion of people estimated to have COPD by age group and gender in England (estimates for 2005)

**Age-group (Years)**	Men Number (%)*	**Women Number (%)**	**Both sexes Number (%)**
15–44	137,530 (1.30)	93,450 (0.89)	230,980 (1.10)
45–54	75,720 (2.38)	64,840 (2.00)	140,560(2.19)
55–64	198,400(6.90)	122,440 (4.11)	320,840 (5.48)
65–74	199,840(10.03)	105,740 (4.81)	305,580 (7.29)
75+	172,700(11.65)	132,400 (5.55)	305,100 (7.89)

Total 15+	784,190 (3.89)	518,870 (2.41)	1,303,060(3.15)
Total 45+	646,660 (6.76)	425,420 (3.92)	1,072,080(5.27)

* Values in brackets indicate age-gender specific prevalence rates (%) of COPD

The assumption that ethnicity is not associated with being a case of COPD, i.e. that all population has the same risk of whites, did not change the total national prevalence estimates considerably (1,297 thousand in 15 year-olds and over and 1,065 thousand over 45s under this assumption). When we considered the risk of COPD in under 35s as equal to the average baseline risk in this age group (in non-smokers), the total number of cases estimated was reduced by 60,800, resulting in an overall prevalence of 1.25 million or 3% (3.8% in men and 2.3% in women). Considering the risk in all over 40s as equal to the average baseline risk, the total number of cases is reduced further to 1,.223,200 (3.0% overall prevalence; 3.7% in men and 2.3% in women). If we consider the risk in under 35s as inexistent, the total number of estimated cases decreases to 1,185,700 (2.9% overall prevalence; 3.4% in men and 2.2% in women). Assuming the more extreme situation of zero risk in all under 40s, the prevalence comes down to 1,128,550 (2.7% overall; or 3.4% in men and 2.11% in women), representing a decrease of 174,500 compared to the original estimates The latter estimates assume that all cases of airflow obstruction in the younger age groups are due to other diagnoses than COPD, such as asthma.

Table 3 shows the estimated prevalences of COPD in urban, suburban and rural England, based on the national population distribution and smoking prevalence. The values in brackets show the estimated average prevalence for areas in the lower and highest quintiles of deprivation. The average prevalence in over 45s varies 4-fold, with the highest values in men in deprived urban areas, and the lowest in women in wealthy rural areas. When the effect of ethnicity is also considered, the variation in prevalence reaches 7-fold, from 1.7% in Asian women from rural areas in the lower quintile of deprivation to 12.5% in black men from urban areas in the upper quintile of deprivation.

**Table 3:** Estimated prevalence P (percent) of COPD in England and according to area of residence

**Area**	**P men age 15+**	**P men age 45+**	**P women 15+**	**P women 45+**
Urban	4.86 (3.65–6.47)	8.41 (6.35–11.12)	3.68 (2.91–4.39)	5.95 (4.71–7.05)
Suburban	3.74 (2.75–4.94)	6.50 (4.79–8.55)	2.23 (1.79–2.69)	3.62 (2.91–4.36)
Rural	3.65 (2.75–4.85)	6.35 (4.80–8.40)	2.05 (1.67–2.53)	3.33 (2.72–4.10)

***England***	***3.89 (2.89–5.16)***	***6.76 (5.04–8.92)***	***2.41 (1.94–2.92)***	***3.92 (3.15–4.72)***

* Values in brackets correspond to mean values in extreme quintiles of deprivation score, or approximately the 10^th ^and 90^th ^percentiles of the prevalence distribution.

## Discussion

We developed a mathematical model that enables the prevalence of COPD to be estimated based on information that is easily available to Primary Care Trusts and many individual general practices. The model takes into account the increasing prevalence of COPD with age and smoking, and the modifying effect of gender. It also considers a higher risk of COPD among those living in urban environments and in areas of higher deprivation, and in black ethnicities.

As expected, the risk associated with smoking and former-smoking is higher in men compared to women. This may be related to their longer history and intensity of smoking, as compared to women. The effects of ethnicity and area of residence are more evident in women, among whom deprivation score is not apparently relevant, after other variables are considered. Urban environment increases the risk of COPD, possibly through higher air pollution levels. Social deprivation may increase the risk of COPD through complex mechanisms in addition to the higher prevalence of smoking. This may include different smoking habits (the model does not take into account duration and intensity of smoking as such information is not readily available), and a higher likelihood of exposure to other risk factors, which are not easily measured, such as passive exposure to tobacco smoking, history of respiratory infections, and less access to health services and information. Ethnic differences in susceptibility are less clear and less well understood, but might involve a combination of behavioural, environmental and possibly genetic factors.

We estimated the overall prevalence of COPD in England as 3.1% in people over 15 years old and 5.3% in those over 45 years old. The model illustrates the huge inequalities in the prevalence of COPD across England, with extreme risks in black men in urban deprived areas in one end of the risk spectrum, and Asian women in the lowest deprived rural areas, in the opposite end, between whom the risk of COPD varies 7-fold on average. Thus simpler models that do not take into account such variations in prevalence across population groups, or the extrapolation of national COPD prevalence figures for smaller areas, would be inappropriate for local use.

A systematic review of good quality COPD prevalence studies yielded estimates for England of between 4% and 10% [17]. The Health Needs Assessment report suggests a prevalence of 5% for men and 3% for women of middle age and upwards [18]. Our results are similar to the latter, but not as high as suggested by some of the studies used for the former review. The decreasing trend in smoking prevalence in England is likely to lead to slow reductions in the real prevalence of COPD. However, major causes of variations in estimates include differences in the populations and in the diagnostic criteria used [19]. This is illustrated by the finding of a prevalence of 13.3% in over 35s in the HSE survey, when a different definition of COPD was used [20]. That study also calculated the prevalence directly from the survey data, differently from our study, where estimates were extrapolated for the population of England.

The figures estimated by the Model-HSE for England are in general slightly lower than, but comparable with other studies on COPD using the same BTS definition, i.e. 4.5% in Norway [21], 6.8% in the US [22] and 6.8% in white males 40–60 years old in Spain [23]. They are also similar to the overall prevalence of 6.1% found in the NICECOPD study for Belfast white population aged 40 to 69 years [24]. The slightly lower estimated prevalence in our study may be largely explained by the lower smoking prevalence in England, but also by differences in the study populations, and the larger study size of the Health Survey for England.

Comparisons of our results with studies that used other definitions of COPD are difficult to interpret. Estimates based on self-reported symptoms tend to overestimate the prevalence. This is because diagnostic specificity is reduced as other respiratory diseases may be misdiagnosed as COPD, although asymptomatic cases of airflow obstruction will be missed. On the other hand, medically diagnosed COPD tends to under-estimate the true prevalence of the disease, as diagnostic sensitivity is reduced. Compared to other commonly used COPD spirometric-based case definitions, the BTS is based on quite conservative cut-off points, yielding relatively low estimate values [10,17].

A main advantage of the Model-HSE is that it is based on high quality data from a large representative sample of the population, and uses standard and specific diagnostic criteria for COPD, which is based on lung function rather than symptoms. Response rates were high in the survey with the achieved samples matching the target populations closely [25]. Prevalence estimates are based on the strength of association between key risk factors for COPD, including the effects of ethnicity, area of residence and deprivation, which were shown to be independent risk factors for COPD in the HSE survey. This represents a significant advantage in relation to previous COPD prevalence models [8,9], which were based only on smoking status, age and gender (also used in the Model-HSE) of mostly white populations outside the United Kingdom. The input data are usually readily available at local level.

The model uses current smoking status (never, former, current) as surrogates for total exposure to cigarette smoke, and is therefore not ideal to predict short term effects of changes in smoking prevalence (e.g. following intervention), due to long latent periods, large time lag between intervention and effects, and irreversibility of disease. However, the model is not static and will be updated over time, as parameters' values change, e.g. smoking prevalence (in England there are regular estimates of smoking prevalence at sub-regional level). Noteworthy, since intensity and duration of smoking (and thus smoking associated risk of COPD) tend to be lower in younger populations [25], the model may slightly overestimate the prevalence in young people. Another reason for prevalence overestimation in young ages is a possible misclassification of cases of asthma into COPD (note reversibility test was not used in the HSE). We dealt with these by providing alternative estimates for the prevalence of COPD, applying baseline or nil prevalence rates in all those under 35 and under 40 years old. These brought the overall prevalence estimates down by 0.1% to 0.4%. The model also relies on the quality of smoking and other data, and does not take into account competing causes of morbidity and mortality in the population, e.g. cardiovascular disease and lung cancer, which may affect prevalence of COPD. Moreover, it still needs correction for populations with significant occupational exposures. There is some degree of imprecision in the estimates, which are larger when the rates are estimated for smaller populations or sub-groups, such as ethnic minorities and specific age-groups. Therefore we recommend that it is used primarily to derive overall population prevalence estimates, rather than estimates within population sub-groups. Further validation with a representative sample of the UK population including large proportions of people from ethnic minorities is still needed, before it may be reliably used in small population groups, such as GP practices and in ethnic minorities. We are currently planning to validate the model at practice level in North West London and investigating the feasibility of two COPD case-finding strategies.

Respiratory function indices have been shown to be predictive of mortality from respiratory disease, cardiovascular disease and all cause mortality [26,27]. Airflow limitation may precede the development of significant symptoms of COPD by many years and its progression is directly linked to the continuing exposure to risk factors, particularly tobacco smoking. As COPD is difficult to diagnose clinically (without spirometry) in its milder forms, it is often diagnosed late – the average age at diagnosis of COPD in the UK is 67 years [28].

Widespread use of spirometry allowing early detection of airflow obstruction has been increasingly advocated as it enables early management of COPD [29]. Stopping smoking prevents the development of COPD, or slows its progress and reduces the risk of hospital admissions [30]. Smoking cessation programmes are highly cost-effective, and crucially, have been specifically shown to be cost-effective when directed to individuals with asymptomatic airway obstruction [31]. This is because smokers may be motivated to attempt to quit when given a diagnosis of airflow limitation [32]. The incremental cost effectiveness ratio (ICER) of opportunistic COPD case-finding for this purpose is a cost per life year gained of £713.16 and a cost per QALY of £814.56 4 [1].

The magnitude of undiagnosed cases can be ascertained by comparing the model estimates with the recorded prevalence of COPD, to indicate the extent of unmet needs in COPD. In the UK this is facilitated by GP performance payments for COPD management through the Quality and Outcomes Framework (QOF) of the GP Contract based on an electronic register of all patients with diagnosed COPD. If this is linked to case finding and intervention, there is a potential for reducing the population burden and progression of the disease.

The average QOF-diagnosed prevalence of COPD in England reported in 2004–5 through the Quality and Outcomes Framework of the GP Contract was only1.4% [33]. This indicates that around 600,000 or nearly half of the 1.3 million COPD cases remain undiagnosed. A relatively large number of these individuals live in London or North of England (data not shown). Many of such cases will continue their risk behaviours and eventually present to the health services at later stages as more severe cases, possibly through emergency hospital admissions. Many of them will become high intensity users of health services, with considerable costs to individuals, the NHS and society. An audit of 80,000 COPD admissions showed that 70% of them are of patients not previously admitted with the condition (Bird M, personal communication).

The Model-HSE may be freely obtained directly from the authors and is publicly available on the Eastern Region Health Observatory website [34]. It may be used, with the qualifications stated above, by general practices and primary care trusts (PCTs), in England, and indeed in other user defined populations in the country and probably in other countries of the UK i.e. Wales, Scotland and Northern Ireland. General practices can use their own data on the population distribution by age-group and gender, smoking prevalence, ethnicity, degree of urbanisation and deprivation score for the area where the practice is located, to obtain indicative figures on the prevalence of COPD in their population. Data from the Primary Care Trust where the practice is located may be used as a proxy (with any relevant adjustments) in cases where practice specific data are not available. In addition it may be valid in Western populations that are not too dissimilar from England.

## Conclusion

We believe that compared to previous models and prevalence estimates, the HSE-Model offers the most reliable estimates for England and the United Kingdom. It recognises deprivation, urban living and ethnicity as independent risk factors for COPD, which are taken into account in the estimates derived, in addition to smoking, age and gender. The model gives prevalence estimates for areas of varying sizes, including large populations at local level, however, the precision of the estimates will be higher for larger areas.

The overall prevalence of COPD in England is estimated as 1.3 million, of whom as many as 600,000 people may be unaware of their diagnosis, therefore missing the opportunity of benefiting from early interventions. This emphasises the importance of active case finding and the model can be used to identify areas with a high level of unmet needs, i.e. with a high proportion of undiagnosed disease, where the benefits of case finding would be optimised. This strategy may also have an impact on reducing health inequalities, due to the socio-economic class gradient in COPD prevalence. The model should be validated, and case-finding strategies using the model should be evaluated for their cost-effectiveness.

## Competing interests

The author(s) declare that they have no competing interests.

## Authors' contributions

LN was responsible for the conception and design, analysis and interpretation of data, and drafted the article. MJ contributed to conception, design and interpretation of data and with critical revision for important intellectual content. TM contributed to analysis and interpretation of data and revisited it critically for important intellectual content. All authors read and approved the final manuscript.
